# Molecular Docking and Molecular Dynamics Study of Propolis Compounds of Sulabiroin-A, Sulabiroin-B, and Broussoflavonol F Toward Tuberculosis 3PTY Target Protein

**DOI:** 10.1155/jotm/6631193

**Published:** 2025-04-30

**Authors:** Jaka Fajar Fatriansyah, Agrin Febrian Pradana, Anggit Driasaditya, Aditya Asprilla Sinaga, Muhamad Sahlan, Siti Norasmah Surip

**Affiliations:** ^1^Department of Metallurgical and Materials Engineering, Faculty of Engineering, Universitas Indonesia, Depok 16424, Jawa Barat, Indonesia; ^2^Department of Chemical Engineering, Faculty of Engineering, Universitas Indonesia, Depok 16424, Jawa Barat, Indonesia; ^3^School of Industrial Technology, Faculty of Applied Sciences, Universiti Teknologi MARA, Shah Alam 40450, Selangor, Malaysia

**Keywords:** 3PTY, molecular docking, molecular dynamics, propolis, tuberculosis

## Abstract

Molecular docking and molecular dynamics simulations were conducted to assess propolis compounds of sulabiroin-A, sulabiroin-B, and broussoflavonol F as tuberculosis (TB) inhibitors with rifampicin as the control ligand. TB remains a significant world health concern, requiring the development of new drug candidates to address more drug-resistant variants. The target protein chosen was 3PTY. The molecular docking simulation showed that sulabiroin-A, sulabiroin-B, and broussoflavonol F docking scores are comparable to rifampicin, with the order of docking score from least favorable to more favorable is sulabiroin-B< sulabiroin-A< rifampicin< broussoflavonol F (−3.397, −3.449, −5.256, −5.961). Molecular dynamics simulations also demonstrated that sulabiroin-B exhibited stable interactions with the target protein, comparable to rifampicin, while sulabiroin-A and broussoflavonol F demonstrated increased fluctuation, suggesting possible instability. Absorption, distribution, metabolism, excretion, and toxicity (ADMET) study verified that all three drugs possess advantageous pharmacokinetic characteristics, with broussoflavonol F exhibiting the most favorable safety and tolerability profile. According to these findings, sulabiroin-B is recognized as the most promising candidate for TB treatment owing to its enhanced stability in molecular dynamics simulations, although broussoflavonol F and sulabiroin-A exhibit intermediate promise. Additional experimental validation is advised to verify their therapeutic efficacy.

## 1. Introduction

In 2019, tuberculosis (TB), a transmissible infection, was positioned among the 10 driving causes of mortality in low-income nations [1–4]. *Mycobacterium tuberculosis*–caused (pulmonary) TB induces symptoms such as severe coughing, tiredness, fever, sweating at night, and even loss of appetite, which causes weight loss. Without proper treatment, TB is a very dangerous disease, and almost 70% of infected people die less than 10 years after the first known contraction [5]. The emergence of COVID-19 in 2019 may exacerbate the spread of TB strains of *Mycobacterium tuberculosis* [6, 7].

TB can appear clinically as “latent” TB or active TB, and the therapy for both is different. Isoniazid, rifampicin, ethambutol, and pyrazinamide are four first-line drugs which is recommended for 6 months. With the proper administration, the success rate reaches 85% [8]. Isoniazid was approved by the Food and Drugs Administration (FDA) [9] for excellent displaying early bactericidal activity (EBA), which is suitable for latent TB. In contrast, rifampicin's excellent sterilizing activity is suitable for active TB [10]. In any case, the treatment of TB could be delayed, and it carries the chance of unfavorable impacts as well as potential intelligence between complex restorative regimens. Tragically, these variables contribute to the rise of multi–drug-resistant (MDR-TB) and extensively drug-resistant (XDR-TB) strains [11].

The treatment of TB could be a drawn-out handle, including complex drug regimens that are regularly related to unfavorable impacts, drug intelligence, and destitute patient adherence. Thus, this has contributed to the rise of MDR-TB and XDR-TB strains [12]. Although medical disclosure and advancement endeavors are continuous, the method remains long and challenging in terms of the advancing nature of TB. The essential objective is to create anti-TB drugs that successfully target MDR-TB and offer shorter treatment lengths [13]. There is an urgent need to discover and develop new anti-TB therapies, mainly aimed at combating drug-resistant and dormant strains of *Mycobacterium tuberculosis* while also reducing treatment duration.

Natural and herbal drugs and/or remedies have been utilized extensively in medicine for centuries. These drugs constitute a highly diverse collection of exceedingly heterogeneous compounds that have developed to interact exclusively with biological targets and are essential for the development of pharmaceutical drugs [14]. Propolis, a bee glue, is a natural compound produced by stingless and honeybees, which bees use to repair their mouths, smoothing their hives, and antiseptic coating to protect their health [13]. Propolis may contain polyphenols, terpenoids, steroids, and amino acids, and its chemical composition is highly varied depending on the region [15].

Propolis produced from stingless bees (Tetragonula aff. biroi) from Sulawesi Island, Indonesia, is known to have sulabiroin-A, isorhamnetin, and glyasperin A [16]. These three compounds have been investigated through the in silico method to potentially inhibit the binding of ACE-2 for SARS-COV-2 [17, 18]. Zulhendri [19] also supported experimental evidence that propolis can treat respiratory tract-related diseases and disorders. Mahani [20] studied the efficacy of propolis supplementation for pulmonary TB patients and found that propolis is beneficial in accelerating treatment effects and recovery [21, 22].

*In silico* methods, molecular docking, and dynamics are powerful computational methods to predict the inhibition activity of a compound or ligand toward a target/protein, and they are becoming more important in drug discovery [23–25]. The results show that a binding score can predict the affinity between ligand and target, which can help in vitro and in vivo experimentalists narrow down the choice of ligands or develop new compounds and drugs.

In this study, molecular docking and dynamics simulation were performed to examine the potential of the propolis compound of sulabiroin-A, sulabiroin-B, and broussoflavonol F as TB inhibitors with the control ligand of rifampicin. The target protein used is 3PTY, as also examined by Kumar [26], but with different ligands. 3PTY is representative for the minimized structure of the C-terminal domain of arabinosyltransferase C (EmbC) enzyme. EmbC, a transferase enzyme protein that specifically exists in the extracellular domain of *Mycobacterium tuberculosis*, is the causative agent of TB disease. EmbC is critical in many biological processes of *Mycobacterium tuberculosis*. It contributes to producing a crucial component of mycobacterium's cell wall. The participation of arabinosyl units in the fusion of diverse cell wall components significantly impacts the biosynthesis process of mycobacterial cell walls [27]. Thus, inhibiting the EmbC enzyme could hinder the cell wall synthesis, which is crucial for mycobacterium's existence. Ali [13] also screened propolis compounds to obtain docking scores. However, our study extended to the molecular docking method and molecular dynamics to observe dynamic interaction between ligand and target. This research aims to assess the potential of sulabiroin-A, sulabiroin-B, and broussoflavonol F propolis compounds as TB drugs.

## 2. Computational Methods

Molecular docking was conducted utilizing the glide docking function of the Maestro Schrodinger software. Then, utilizing an NPT ensemble and the Desmond function of the Maestro software, a molecular dynamics (MD) simulation was performed for 100 nanoseconds with a trajectory record of 10 picoseconds (ps). This study utilized the Maestro Schrödinger Software to simulate molecular docking and molecular dynamics. The pk CSM and Molsoft websites provided toxicology predictions for ligands and ADMET.

### 2.1. Ligand and Protein Preparation

**Figure 1:**
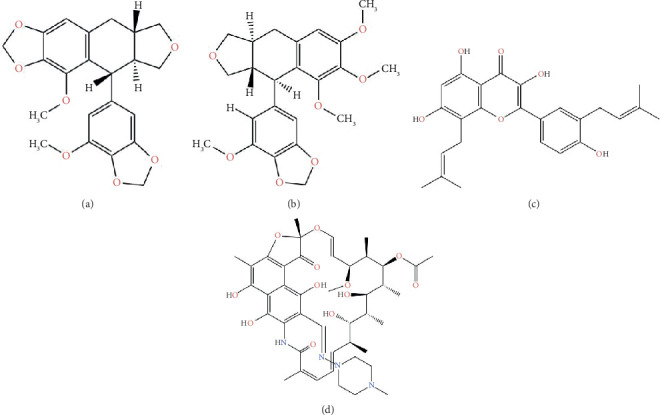
The structure ligands of (a) sulabiroin-A, (b) sulabiroin-B, (c) broussoflavonol F, and (d) rifampicin.

This study utilized the PubChem database to acquire the three-dimensional structure of sulabiroin-A, sulabiroin-B, broussoflavonol F, and rifampicin ligands. The structure of all ligands is shown in Figures 1(a), 1(b), 1(c), and 1(d). The three-dimensional structures of each ligand were initially prepared using the LigPrep function of the Maestro application. During the process of preparation, the elimination of hydrogen atoms occurs. Epik v2.9 was used to analyze the ionization states of ligands and tautomers at pH 7 ± 2. The force field employed was OPLS3e, and the optimal structure with the lowest ionization penalty was selected.

Meanwhile, the three-dimensional conformation of the selected receptor protein arabinosyltransferase C (EmbC) was acquired from the protein data bank (PDB) website under the accession number PDB ID 3PTY [28]. The resolution of protein structure was 2.0 A, which was acquired from X-ray diffraction data. The Maestro software's protein preparation wizard function was used to create the three-dimensional conformation of proteins easier by attaching hydrogen atoms, removing noninteracting water molecules, improving protein structure, and optimizing hydrogen bonding within proteins.

### 2.2. ADMET Prediction

The drug-likeness of the ligand compounds was determined using the molecular properties of each compound, Lipinski's rule of five, and the drug-likeness model score. The former and the latter were retrieved from the websites of https://molsoft.com/mprop/ and https://biosig.lab.uq.edu.au/pkcsm/prediction (pkCSM) [29], respectively.

### 2.3. Molecular Docking

The grid area was defined using the receptor grid generation feature, identifying the specific region within the system where the receptor operates. This grid was created with the Maestro software's receptor grid generation tool, and it was centered on the protein's natural ligand site, located in the active site. The grid coordinates were *X* = 94.27, *Y* = 10.72, and *Z* = 3.82, with a box size of 20. Molecular docking was conducted using the Glide docking tool, with the van der Waals radius set to 1 and the partial charge cutoff at 0.25 in the ligand section. The docking utilized an extra precision (XP) setting, with tethered ligands meeting criteria such as having more than 500 atoms and over 100 rotatable bonds. To ensure that docking scores are reliable, the validation was conducted by redocking and calculating docking scores using a different software, AutoDock Vina [30]. The docking scores were calculated a minimum of five times and no significant results were observed, and the comparison with the docking score obtained by AutoDock Vina was consistent with our software.

### 2.4. Molecular Dynamics Simulation

The molecular dynamics simulation utilized of the docked complex was obtained using XP Glide docking. The system builder function is configured by specifying the solvent using the Simple Point Charge (SPC) water model, shown as a cubic simulation box. The simulation box was determined using the buffer approach, with the distances a, b, and c fixed at 10 Å. To avoid ions and salts that were 20 Å apart, the next step was to reduce the solvent volume in which the ions section was positioned. 4Na + ions and a 0.15 M salt solution were added to complete the neutralization. The cutoff radius interaction was 9 Å. The developed system was then prepared for molecular dynamics simulation using the desmond molecular dynamics method. The NPT ensemble was utilized to run a molecular dynamics simulation for 100 ns with a 10-ps trajectory. The temperature was set to 310 K using the Nosé–Hoover thermostat, and the pressure was set to 1.01325 bar using Martyna–Tobias–Klein. The NPT ensemble was used to resemble the pressure effect of the biological system, though the initial or equilibrium state was achieved using the NVT ensemble. The stability of ligand–protein interaction was analyzed using root mean square deviation (RMSD), root mean square fluctuation (RMSF), and protein–ligand contact parameter results.

## 3. Results

### 3.1. ADMET Prediction

Table 1 presented the toxicology prediction results, including the predicted values based on Lipinski's rule of five and the Druglikeness score. These results provide an overview of the compound's viability in drug development, considering the ideal pharmacokinetic characteristics for absorption, distribution, metabolism, and excretion. The data in the table are then reviewed using the theoretical framework provided on the pkSCM website to assess absorption, distribution, metabolism, excretion, and toxicity shown in Table 2.

### 3.2. Molecular Docking Results

Molecular docking simulations were conducted for each ligand. The molecular docking score, also known as the docking score, was obtained. The data acquired from the molecular docking simulation are presented in Table 3.

### 3.3. Molecular Dynamic Results

**Figure 2:**
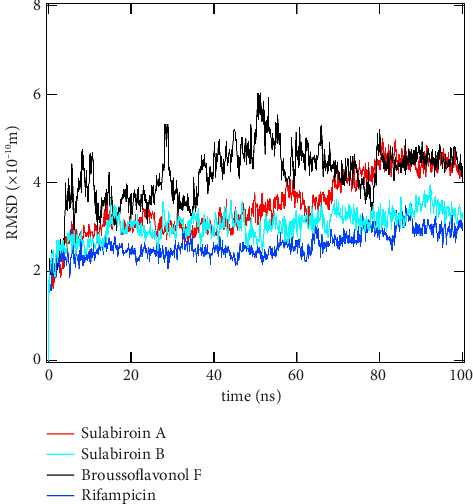
RMSD plots for all ligands: sulabiroin-A, sulabiroin-B, broussoflavonol F, and rifampicin (control ligand).

**Figure 3:**
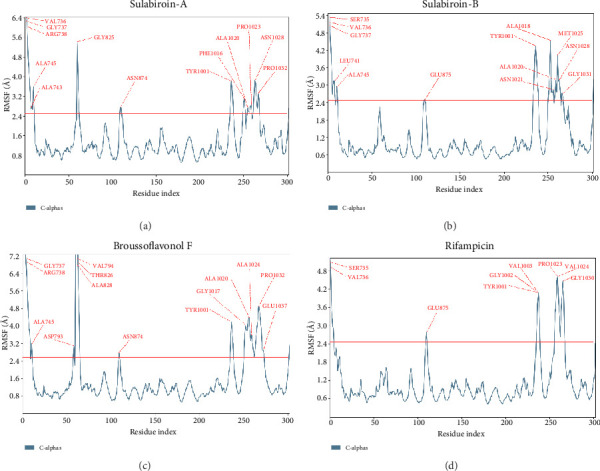
RMSF plots for each ligand: (a) sulabiroin-A; (b) sulabiroin-B; (c) broussoflavonol F; (d) rifampicin (control ligand).

**Figure 4:**
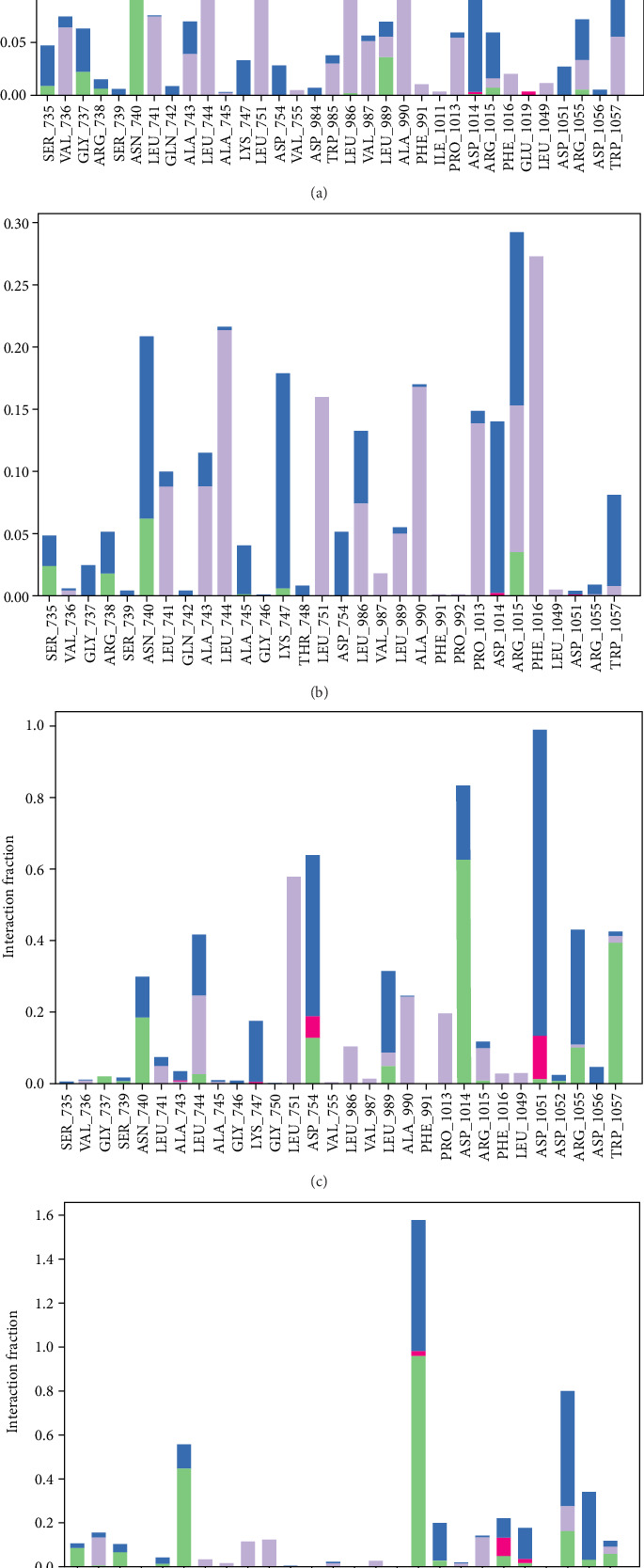
The simulation interaction of each ligand with the protein target: (a) sulabiroin-A; (b) sulabiroin-B; (c) broussoflavonol F; (d) rifampicin (control ligand).

Molecular dynamics simulation studies were used to verify the stability of docked complexes and the binding pose found in docking investigations. RMSD, RMSF, and protein–ligand contact are the results of the molecular dynamic simulation shown in Figures 2, 3, and 4.

## 4. Discussion

### 4.1. ADMET Prediction

The ligand toxicology test results, including Lipinski's rule and drug feasibility scores, are displayed in Table 1. All ligands in Table 1 comply with Lipinski's rule except for rifampicin, a control ligand, and thus, it is categorized as beyond the rule of 5 (bRo5). However, Machado et al. [27] commented that rifampicin is exempted from rules due to its use as a repurposed drug and its use at higher doses. On the other hand, as first-line TB medicines, isoniazid, ethambutol, and pyrazinamide completely adhere to Lipinski's rule, suggesting excellent drug-like qualities. The propolis compounds sulabiroin-A, sulabiroin-B, and broussoflavonol F all meet Lipinski's criteria, with the exception of broussoflavonol F, which has one violation (log *p* = 5.61 > 5). Lipinski's criteria are not the sole determinant in evaluating the suitability of a molecule for therapeutic application [31]. Although numerous substances do not adhere to the Lipinski criteria and are anticipated to exhibit limited bioavailability, they are commonly employed as pharmaceuticals in the commercial sector [28]. The limits of Lipinski's principles need a more thorough evaluation of pharmacological feasibility, notably in predicting ADMET features. Table 2 provides additional information on the ADMET forecast. The data in the table are then tested using the theory described on the pk CSM website.

#### 4.1.1. Adsorption

All ligands are fairly water-soluble, particularly sulabiroin-A and sulabiroin-B, which are moderately soluble, and broussoflavonol F and rifampicin, which are soluble, qualifying them for drug use and allowing for facile dissolving in the body [32, 33]. The three drug candidates—sulabiroin-A, sulabiroin-B, and broussoflavonol F—are still within the range (−6 to 0) for being accepted as drugs in terms of how well they are absorbed and whether they dissolve in water. Among the first-line medications, isoniazid (−1.6), ethambutol (−0.443), and pyrazinamide (−0.615) show better solubility, making them easily dissolvable in the body, a significant criterion for oral administration.

The three medicine candidates have very high levels of intestinal absorption. Sulabiroin-A and sulabiroin-B reach 100%, and broussoflavonol F shows 90.043% compared to the standard drug rifampicin. This makes all three candidates very good. These findings imply that propolis components and first-line medicines (except rifampicin) have superior bioavailability and absorption efficiency, outperforming rifampicin in this regard.

#### 4.1.2. Distribution and Bioavailability

The volume of distribution (VDss) measures medication dispersion in body tissues. Broussoflavonol F (log VDss = −0.15) and pyrazinamide (−0.338) have the lowest distribution potential, indicating a more restricted and targeted action with less danger of systemic toxicity or protracted clearance. Isoniazid (−0.352) and ethambutol (0.166) have moderate to low distributions, but sulabiroin-A (−0.006) and sulabiroin-B (0.018) are nearly neutral. In contrast, rifampicin (2.185) has a wide tissue distribution, which may result in slower clearance and a higher risk of side effects. The fraction unbound in plasma reveals additional differences: Sulabiroin-B and broussoflavonol F have low unbound fractions (0 and 0, respectively), indicating high protein binding, whereas isoniazid (0.728), ethambutol (0.487), and pyrazinamide (0.773) have higher unbound fractions, indicating greater plasma availability.

#### 4.1.3. Metabolism and Drug Interactions

In terms of metabolism, all drug candidates are substrates for CYP3A4, the enzyme responsible for drug metabolism. However, sulabiroin-A and sulabiroin-B are also acting as inhibitors, potentially affecting the effectiveness and safety of drug use on metabolism and may lead to drug–drug interactions within the cytochrome P450. This found necessitates further pharmacokinetic studies to assess the compound impact on metabolic stability and drug clearance time and rates.

Regarding drug elimination, sulabiroin-A exhibited slower total clearance, while sulabiroin-B and broussoflavonol F demonstrated faster elimination rates. This finding suggests careful dosage requirements. Rifampicin as a control ligand displayed slower elimination, which is consistent with its known clinical application. Broussoflavonol F, rifampicin, isoniazid, ethambutol, and pyrazinamide do not inhibit CYP3A4, indicating a lesser risk of metabolic interference. None of the ligands are CYP2D6 substrates or inhibitors, implying that they have no effect on this metabolism.

#### 4.1.4. Excretion

Total clearance rates differ among ligands. Sulabiroin-A (−0.07) and rifampicin (−0.558) have slower clearance, requiring careful dosage changes. Sulabiroin-B (0.105), broussoflavonol F (0.285), isoniazid (0.722), ethambutol (0.459), and pyrazinamide (0.666) all have faster elimination rates, indicating effective clearance from the body. None of the chemicals are renal OCT2 substrates, implying that renal excretion mechanisms are not considerably altered.

#### 4.1.5. Toxicity and Drug Safety

None of the propolis components or rifampicin cause Ames, hepatotoxicity, or skin sensitivity. However, isoniazid exhibits Ames toxicity, indicating potential mutagenicity, despite remaining a frequently used TB medication with manageable risks. Broussoflavonol F had the greatest maximum tolerated dose (0.603 log mg/kg/day), followed by pyrazinamide (1.354), isoniazid (1.166), and ethambutol (0.792), indicating greater tolerance than sulabiroin-A (0.076), sulabiroin-B (0.099), and rifampicin (0.253). For oral rat acute toxicity (LD50), sulabiroin-A (2.943 mol/kg) and sulabiroin-B (2.878) exhibit greater values (lower toxicity) than broussoflavonol F (2.562), rifampicin (2.424), isoniazid (2.304), ethambutol (2.482), and pyrazinamide (2.047).

The propolis components sulabiroin-A, sulabiroin-B, and broussoflavonol F have promising pharmacokinetic features, such as high absorption and bioavailability, that are comparable to or superior to the first-line TB medicines isoniazid, ethambutol, and pyrazinamide. Broussoflavonol F stands out for its greater tolerance, low toxicity, and regulated distribution, making it a viable alternative or supplement to rifampicin. Sulabiroin-A and sulabiroin-B, while successful in absorption, require additional research into their metabolic stability due to CYP3A4 suppression. Among first-line medicines, ethambutol and pyrazinamide have good characteristics, with high solubility and quick clearance; however, isoniazid's Ames toxicity should be avoided. These findings emphasize the potential of propolis chemicals in TB treatment; however, preclinical and clinical investigations are required to evaluate their therapeutic efficacy and safety in comparison with existing medications.

### 4.2. Molecular Docking

**Figure 5:**
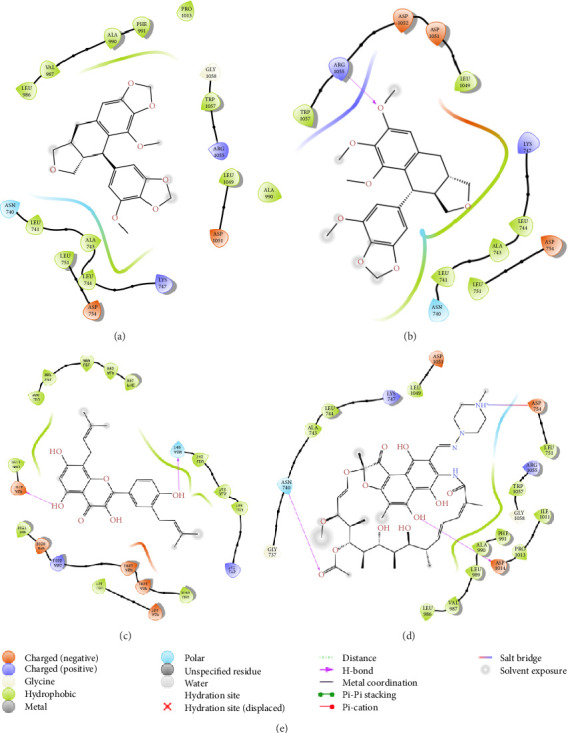
Ligand–protein 2D interaction diagram of molecular docking. (a) sulabiroin-A, (b) sulabiroin-B, (c) broussoflavonol F, and (d) rifampicin (control). (e) Legend of the symbols used in the molecular interaction diagram.

The ligands are sorted based on their molecular docking score and can be seen in Table 3. The target protein and the ligand exhibit a stable interaction, as evidenced by the low docking score. It was found that broussoflavonol F has the most favorable docking score value, followed by rifampicin, sulabiroin-A, and sulabiroin-B. The results show that broussoflavonol F exhibits the strongest binding affinity outperforming even the control ligand of rifampicin. The protein–ligand contact is also shown in Figure 5. The discussion of ligand and protein contact will be discussed in the molecular dynamics subsection.

### 4.3. Molecular Dynamics

#### 4.3.1. RMSD

RMSD is the root mean square displacement of an atom over time. RMSD data show the average distance between atoms, ligands, and proteins.  Figure 2 displays the protein's RMSD relative to ligand plot results. The RSMD data were gathered for 100 ns and measured with the reference of C-alpha (the backbone of the residue/carbon atom of the alpha carbon of each residue). At the beginning of the simulation (0.02 ns), a significant increase in the RMSD value was observed for all ligands, including rifampicin. For 100 ns, only sulabiroin-B and rifampicin show low RMSD values in the range between 1 and 3 Å and both ligands show minimal fluctuation and consistent structural conformation, especially for rifampicin. This result confirms that rifampicin is the strongest and most stable binding to the target while opening the possibility for sulabiroin-B as a drug candidate, which was shown to indicate moderate stability but a higher RMSD than rifampicin.

Broussoflavonol F and sulabiroin-A exhibit very high fluctuation above 3 Å (stable threshold), and especially, broussoflavonol F RMSD reached above 5 Å at certain points. This finding shows that broussoflavonol F's structure is not as stable as it could be, which means that the ligand could reconfigure within the binding site. This large fluctuation is due to weaker interactions and low conformational flexibility. Sulabiroin-A also exhibits high, or rather moderate, fluctuation, and in 100 ns, the fluctuation is like broussoflavonol F, and this result indicates that sulabiroin-A is not as stable as sulabiroin-B as a drug candidate.

#### 4.3.2. RMSF

The RMSF determines the atomic positions' deviation from the point of origin. In simple terms, RMSF demonstrates the dynamic nature of the protein–ligand interaction. Similar patterns can be seen in the ligand–protein RMSF plot for each ligand, with the start and end N-terminal and C-terminal residues having very high RMSF values because they represent the free-moving, highly reactive tails or ends of the protein structure. For every ligand, the RMSF charts are shown in Figure 3. The acceptable value of RMSF is below 2.5 Å [18].

Figure 3 shows the RMSF values for all of the protein residues that interacted with each ligand (a) sulabiroin-A, (b) sulabiroin-B, (c) broussoflavonol F, and (d) rifampicin. Sulabiroin-A has 13 residues with a high RMSF value (RMSF value > 2.5 Å), none of which are in the protein binding site. For sulabiroin-B, the total number of residues with a high RMSF value was three, and none were in the protein binding site. For broussoflavonol F, the total number of residues with a high RMSF value was six, and only residue of ALA 743 was in the protein binding site. Lastly, rifampicin has five residues with a high RMSF value; none were in protein binding sites. These findings show that the interaction between ligand and the active binding site of protein is stable in that none of the residues in the active binding site fluctuated much.

For sulabiroin-A, the interaction is dominated by hydrophobic interaction between sulabiroin-A and nonpolar protein residues of LEU 751, LEU 744, LEU 986, and ALA 990, and they are in or near active binding sites (see Figure 5(a)). There are also hydrogen bond interactions with the polar residues of ASN 740 and SER 735. The domination of hydrophobic interaction does not give strong ligand–target interaction, and it can be seen by high RMSD values in comparison with rifampicin, a ligand control. When Figure 4(a) is compared to Figure 5(a), there is a possible loss of hydrogen interaction and thus makes the interaction slightly weaker over time, and the interaction shifts to hydrophobic interaction, which is not as strong and hydrophobic interaction.

For sulabiroin-B, the interaction is dominated by hydrophobic interaction between sulabiroin-A and nonpolar protein residues of PHE 1016, LEU 744, ALA 990, and PRO 1013. Among these residues, Only LEU 744 and ALA 990 are observed in Figure 5 which suggests that there is possible partial dissociation from protein or shift over time. There are more hydrogen bonds and water bridges formed between ligand and protein in comparison with sulabiroin-A suggesting stronger interaction between ligand and protein. The results are confirmed by Figure 2 which demonstrated that sulabiroin-A has low RMSD values, and comparable to rifampicin.

Broussoflavonol F gives a more balanced interaction between ligand and protein residues by bringing more H-bond interaction and hydrophobic and water bridge interaction. H-bonds are thought to be important in ligand–protein interaction, which brings more affinity and strong interaction [34]. The residue ASP 1014 brings dominant H-bond interaction, while ASP 1051 brings water bridge interaction, and other residues in Figure 4(c) bring combination between the hydrophobic bond and water bridges. This strong interaction also explains that broussoflavonol F has a favorable docking score. However, broussoflavonol F yields very high RMSD. This result attenuates that docking score is not enough to describe ligand–protein interaction.

Rifampicin as a ligand control brings very strong (absolute value) hydrogen bonds and water bridge interaction. The strong hydrogen bonds are also observed in Figure 5. This result is aligned with the relatively high docking score of rifampicin and the low value of RMSD. Overall, only sulabiroin-B gives strong ligand–protein interaction performance, on par with rifampicin, although not close.

Even though our study did not have any in vitro or in vivo experiments, the results of our simulations are very similar to those from Siheri et al. experiment [35] that indicated that Libyan propolis could inhibit Mycobacterium. In their research, it was discovered that Libyan propolis extract has an IC50 value of 53.2 μg/mL. This is less than the IC50 value of 141.5 μg/mL [36] for rifampicin against Mycobacterium, but it is still good.

## 5. Conclusion

The molecular docking and molecular dynamics simulations have been conducted to observe the interaction between sulabiroin-A, sulabiroin-B, and broussoflavonol F propolis compounds (and rifampicin as control ligand) to target protein 3PTY to assess the potential of these ligands as TB drugs. The molecular docking simulation showed that sulabiroin-A, sulabiroin-B, and broussoflavonol F docking scores are comparable to rifampicin, with the order of docking score from least favorable to more favorable, which is sulabiroin-B < sulabiroin-A < rifampicin < broussoflavonol F (−3.397, −3.449, −5.256, −5.961).

The analyses of ligand–protein contact reveal that the broussoflavonol F and rifampicin docking scores are favorable due to the existence of H-bond interaction as well as hydrophobic interaction in ligand–protein interaction, which gives rise to more stable interaction in comparison with sulabiroin-A which is dominated by hydrophobic interaction and sulabiroin-B which is dominated by weak water bridge interaction. Nonetheless, molecular dynamics simulations revealed that sulabiroin-B demonstrated lowest fluctuation, positioning it as a compelling candidate regarding structural stability.

ADMET predictions indicated that all three compounds demonstrate favorable absorption and bioavailability, with broussoflavonol F distinguishing itself through its superior tolerability and reduced toxicity. Although sulabiroin-A and sulabiroin-B exhibit moderate drug-like characteristics, a more thorough examination of their metabolic stability is warranted.

In summary, sulabiroin-B stands out as the most promising candidate for TB treatment, attributed to its reduced fluctuation and enhanced stability observed in molecular dynamics simulations, factors that are essential for sustaining ligand–protein interaction stability over time. Although broussoflavonol F demonstrated a superior docking score, its significant fluctuations in molecular dynamics indicate a lack of stability, thereby rendering sulabiroin-B a more dependable alternative. Sulabiroin-B demonstrates promise owing to its stability observed in molecular dynamics simulations. Subsequent in vitro and in vivo investigations are imperative to substantiate these computational results and evaluate their potential clinical applicability as anti-TB therapeutics.

## Figures and Tables

**Table 1:** The toxicology prediction results include the predicted value of Lipinski's rule of five and the Druglikeness score.

Ligand	Lipinski's rule of five		log P, < 5	Number of hydrogen bond donor (HBD), < 5	Number of hydrogen bond acceptor (HBA), < 10	Molecular weight (MW), < 500	Druglikeness score
	Violation	Druglikeness					
Sulabiroin-A	0	Yes	3.45	0	7	399.14	0.23
Sulabiroin-B	0	Yes	3.72	0	7	414.17	0.44
Broussoflavonol F	1	Yes	5.61	4	6	422.17	0.75
Rifampicin	3	No	4.63	6	14	822.41	1.85
Isoniazid	0	Yes	−0.88	3	3	137.06	0.19
Ethambutol	0	Yes	0.47	4	4	204.18	−0.51
Pyrazinamide	0	Yes	−0.62	2	3	123.04	−0.18

**Table 2:** Prediction results of ADMET properties for ligands.

Properties		Sulabiroin-A	Sulabiroin-B	Broussoflavonol F	Rifampicin	Isoniazid	Ethambutol	Pyrazinamide
Absorption	Water solubility (log mL/L)	−4.439	−4.884	−3.601	−3.008	−1.6	−0.443	−0.615
	Intestinal absorption (%)	100	100	90.043	56.061	92.601	98.414	92.813
	Skin permeability (log *K*_*p*_)	−3.101	−3.048	−2.735	−2.735	−3.351	−2.739	−3.92

Distribution	VDss (log L/kg)	−0.006	0.018	−0.15	2.185	−0.352	0.166	−0.338
	Fraction unbound	0.017	0	0	0.12	0.728	0.487	0.773

Metabolism	CYP2D6 substrate	No	No	No	No	No	No	No
	CYP3A4 substrate	Yes	Yes	Yes	Yes	No	No	No
	CYP2D6 inhibitor	No	No	No	No	No	No	No
	CYP3A4 inhibitor	Yes	Yes	No	No	No	No	No

Excretion	Total clearance	−0.07	0.105	0.285	−0.558	0.722	0.459	0.666
	Renal OCT2 substrate	No	No	No	No	No	No	No

Toxicity	Ames toxicity	No	No	No	No	No	Yes	No
	Max. tolerated dose	0.076	0.099	0.603	0.253	1.166	0.792	1.354
	Oral rat acute toxicity (LD50)	2.943	2.878	2.562	2.424	2.304	2.482	2.047
	Hepatotoxicity	No	No	No	No	No	No	No
	Skin sensitization	No	No	No	No	No	No	No

**Table 3:** Docking scores of all ligands.

Ligand	Docking score (kkal/mol)
Sulabiroin-B	−3.397
Sulabiroin-A	−3.449
Rifampicin	−5.256
Broussoflavonol F	−5.961

## Data Availability

The data that support the findings of this study are available from the corresponding author upon reasonable request.
